# Connecting the solution chemistry of PbI_2_ and MAI: a cyclodextrin-based supramolecular approach to the formation of hybrid halide perovskites†
†Electronic supplementary information (ESI) available: Experimental section; Section 1: solution characterization; Section 2: solar cell optimization and characterization; Section 3: thin film characterization; Section 4: advanced structural characterization. See DOI: 10.1039/c7sc05095j


**DOI:** 10.1039/c7sc05095j

**Published:** 2018-02-12

**Authors:** Sofia Masi, Federica Aiello, Andrea Listorti, Federica Balzano, Davide Altamura, Cinzia Giannini, Rocco Caliandro, Gloria Uccello-Barretta, Aurora Rizzo, Silvia Colella

**Affiliations:** a Istituto di Nanotecnologia CNR-Nanotec , Distretto Tecnologico via Arnesano 16 , 73100 Lecce , Italy . Email: aurora.rizzo@nanotec.cnr.it ; Email: silvia.colella@unisalento.it; b Dipartimento di Matematica e Fisica “E. De Giorgi” , Università del Salento , Via per Arnesano , 73100 Lecce , Italy; c Dipartimento di Chimica e Chimica Industriale , Università di Pisa , Via Moruzzi 13 , 56124 Pisa , Italy; d Istituto di Cristallografia , CNR-IC , Via Amendola 122/O , 70126 Bari , Italy

## Abstract

The evolution from solvated precursors to hybrid halide perovskite films dictates most of the photophysical and optoelectronic properties of the final polycrystalline material. Specifically, the complex equilibria and the importantly different solubilities of lead iodide (PbI_2_) and methylammonium iodide (MAI) induce inhomogeneous crystal growth, often leading to a defect dense film showing non-optimal optoelectronic properties and intrinsic instability. Here, we explore a supramolecular approach based on the use of cyclodextrins (CDs) to modify the underlying solution chemistry. The peculiar phenomenon demonstrated is a tunable complexation between different CDs and MA^+^ cations concurrent to an out of cage PbI_2_ intercalation, representing the first report of a connection between the solvation equilibria of the two perovskite precursors. The optimal conditions in terms of CD cavity size and polarity translate to a neat enhancement of PbI_2_ solubility in the reaction media, leading to an equilibration of the availability of the precursors in solution. The macroscopic result of this is an improved nucleation process, leading to a perovskite material with higher crystallinity, better optical properties and improved moisture resistance. Remarkably, the use of CDs presents a great potential for a wide range of device-related applications, as well as for the development of tailored composite materials.

## Abstract 2

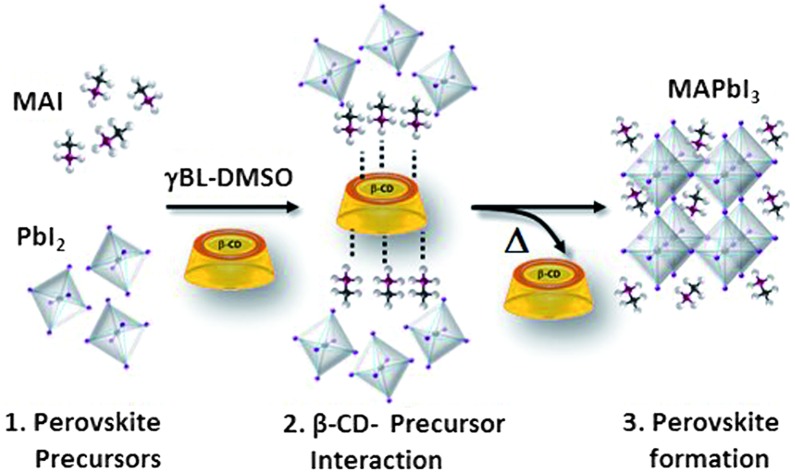
Cyclodextrin macrocycles are able to modify and control the solvation equilibria of hybrid perovskite components in solution by establishing supramolecular interactions.

## Introduction

**Fig. 1:**
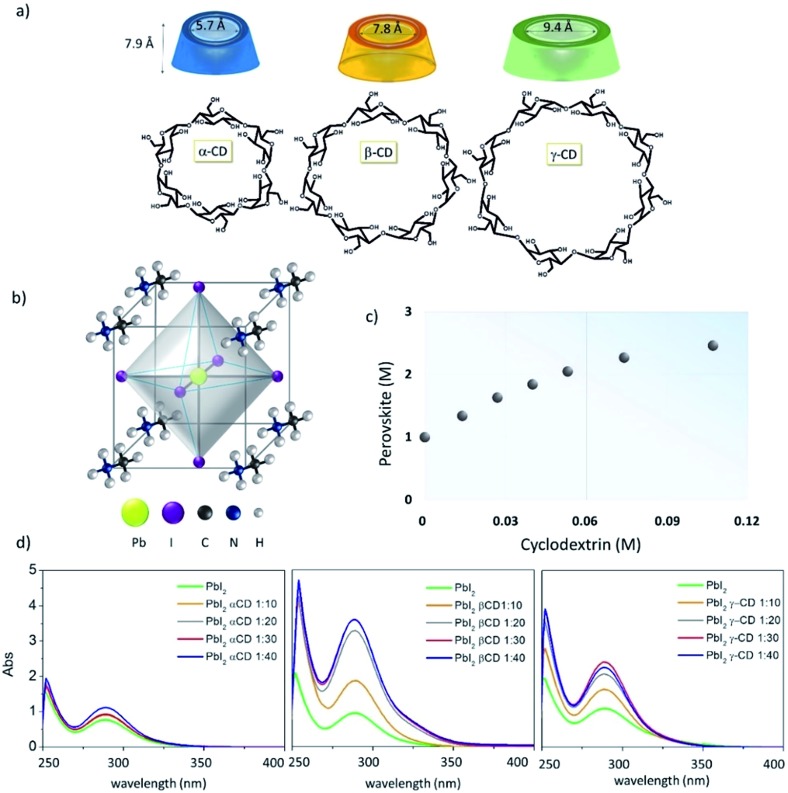
(a) Chemical structures of α-, β- and γ-cyclodextrins; (b) perovskite crystals; (c) a phase solubility diagram of MAPbI_3_ solution with increasing β-cyclodextrin concentration (from 0 M to 0.11 M) in a γ-butyrolactone (GBL) : dimethylsulfoxide (DMSO) 2 : 1 v/v solvent mixture. (d) UV-vis spectra of PbI_2_ in GBL : DMSO 2 : 1, as a function of α-, β- and γ-CD concentration, here illustrated for every 10 equivalents.

Organic–inorganic hybrid perovskites have arisen in previous years as the reference material for many optoelectronic areas.^1^–^3^ They are solids composed of inorganic metal-halide frameworks, filled and neutralized using organic cations in an octahedral arrangement (Fig. 1). The formation of perovskite crystals occurs spontaneously through the self-assembly of the two precursors upon deposition. Such an easy solution processing method suggests budget, large-scale production, which, combined with outstanding optoelectronic properties, makes perovskites attractive for various applications, spanning from lasing to light-emitting diodes^1^,^2^ and solar cells.^3^ In all these fields, the active layer properties are found to be very sensitive to little variations during the film self-assembly process.^4^ The active layer is, in fact, a collection of perovskite crystals loaded with defects, differing in size and orientation, surrounded by an amorphous phase. Logically the optical and electrical properties, along with the stability of these composite films, would dramatically vary with material morphology, crystallinity and defect concentration.^5^ The use of additives, such as polymers,^6^–^8^ fullerenes,^9^,^10^ inorganic acids,^11^,^12^ solvents,^13^–^16^ organic molecules and salts,^17^–^19^ has been widely explored in MAPbI_3_ film formation and has helped in increasing the reproducibility and the morphological control, as well as in improving the stability of perovskite materials. However, one of the most important parameters resulting in poor control over the MAPbI_3_ self-assembly process is generally underestimated or not specifically addressed in these approaches. It is the inhomogeneous crystal growth which originates from severe differences in the solubility of the precursors, being very low for PbI_2_ compared to that for methylammonium iodide.^20^ We propose a novel and general approach to fill this gap, increasing and equilibrating the availability in solution of perovskite precursors through the use of native cyclodextrin macrocycles (CDs) (Fig. 1a). The choice of this class of macromolecule, composed of a three-dimensional truncated cone externally decorated with hydroxyl groups, is motivated by their unique – and proven – ability to establish multiple interactions with a wide variety of chemical species,^17^,^21^–^26^ with the precise aim of forming a supramolecular network in solution simultaneously involving both perovskite precursors. Furthermore, with respect to other macrocyclic hosts, they are commercially available and inexpensive. Here we explore CDs with different cavity sizes (α-, β- and γ-CD, Fig. 1a) and, taking advantage of the solubilizing and nucleating characteristics of β-CDs, we achieve a more than two-fold enhancement of the perovskite precursor solubility limit in the reaction media. We obtain a 2.5 M concentrated solution which is a very viscous ink ideally suited for large area printing and is very stable over time. In this paper we elucidate the chemical-physical mechanisms related to the enhanced solubility and improved nucleation process of the precursors by mainly focusing on the supramolecular assembly between CDs, MAI and PbI_2_. In particular, we explore their interactions in solution using UV-vis absorption and nuclear magnetic resonance (NMR) spectroscopies, and their effect on the perovskite material by advanced structural characterization, with synchrotron X-ray powder diffraction (XPD)/pair distribution function (PDF). We verify that the use of CDs provides the unique, double advantage of simultaneously complexing the organic cation and dissociating PbI_2_ aggregates by intercalation forming more soluble species. Importantly, the solubilizing effect of CD allows improved crystallinity and moisture resistance of the perovskite films to be achieved, whilst still preserving their excellent electrical properties.

## Results and discussion

### Complexation phenomena in solution

The first evidence of the CD effect in solution is the significant enhancement of the perovskite precursor's (MAI : PbI_2_) solubility. In detail, the solubility of the MAI : PbI_2_ blend increases upon increasing the concentration of β-CD, reaching a maximum value of 2.5 M (Fig. 1c), far higher than the 1 M solubility limit in γ-butyrolactone : DMSO (2 : 1).^20^,^27^ Remarkably, we observe the stability of these high concentration solutions for months. In contrast, upon the addition of γ-CD and α-CD we observe a maximum concentration of 2 M and 1.7 M, respectively. The room temperature (RT) solubility of the pristine PbI_2_, the limiting factor of the overall PbI_2_:MAI mixture concentration, is also enhanced by β-CD addition, reaching a value of 1.8 M that is almost doubled compared to the 1 M concentration (in hot solution) obtained without β-CD (Fig. S1a†). For γ- and α-CDs, the PbI_2_ concentration reaches 1.5 M and 1 M, respectively. These findings suggest that in the presence of different CDs the equilibria of ionic species in the MAI : PbI_2_ mixture, such as PbI_2_ complexes (PbI_6_^4–^, PbI_3_^–^ or PbI_4_^2–^) and MA^+^ cations, are strongly altered, possibly promoting the formation of more soluble species and the consequent aggregate consumption, as suggested by experimental observations. The ability of α-, β- and γ-CDs to form a complex with PbI_2_ is investigated by observing the spectral changes of a diluted PbI_2_ solution upon titration with different organic cavities, as shown in Fig. 1d. UV-vis absorption spectra show no significant changes for the PbI_2_/α-CD system, whereas an impressive increase in the PbI_2_ absorption band centered at *λ* = 280 nm is detected with the incremental addition of β-CD, while the trend observed for γ-CD lies in between, in line with the above discussed PbI_2_ solubility enhancement. It is noteworthy that the spectral band position remains practically unaltered while increasing in intensity. We ascribe the observed hyperchromicity to the intercalation of CD between PbI_2_ platelets, originating from the propensity of CDs to complex metal-halides.^28^,^29^ β-CD seems to form soluble complexes with the metal halide allowing, at the macroscale, an increase in PbI_2_ solution concentration up to 1.8 M (Fig. S1a†) and, in particular, solubility of the perovskite precursors up to 2.5 M (Fig. 1c). The intercalation mechanism is evidently less efficient for α-CD, possibly because of its intrinsic lower solubility in the solvent mixture,^30^,^31^ and this eventually leads to less soluble CD–PbI_2_ complexes.^32^ Importantly, the 2.5 M concentrated β-CD based solution exhibits a much higher shear viscosity with respect to the CD-free 1 M solution (Fig. S1b†), making it an ideal candidate for large area deposition techniques in which the ink is subjected to high mechanical stress, such as screen-printing and roll-to-roll.^33^

**Fig. 2:**
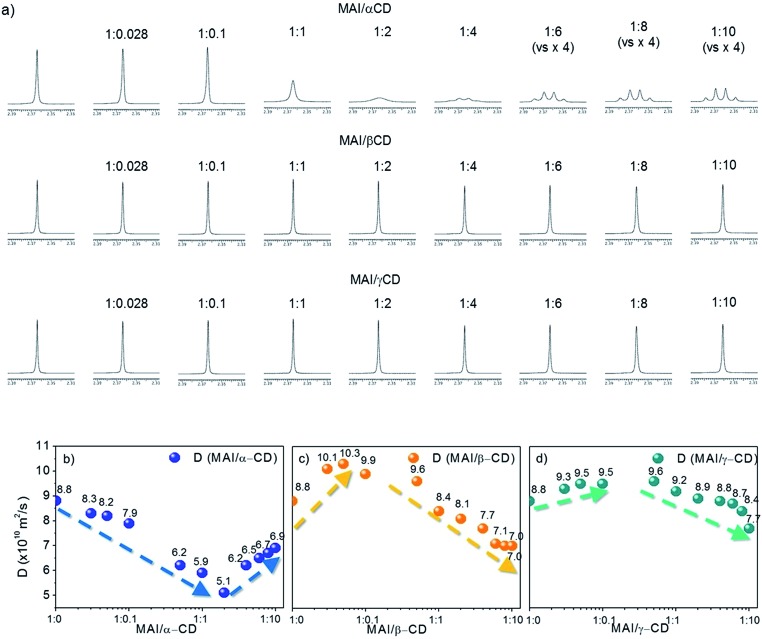
(a) ^1^H NMR (600 MHz, DMSO-d6, 25 °C) spectra of MAI (5 mM) in mixtures with α-CD, β-CD and γ-CD at different molar ratios; expansion of the low-frequency spectral region (from 2.3 ppm to 2.4 ppm). Plots showing the variation of diffusion coefficient (*D*) of MAI during titration with (b) α-CD, (c) β-CD and (d) γ-CD.

To shed light on the mechanism leading to the improved solubility and the possible additional effects involving weak interactions between MAI and the CD macrocycles, we investigate our systems using solution NMR spectroscopy. ^1^H NMR spectra and DOSY maps are recorded for binary mixtures of MAI with α-, β- or γ-CD at different molar ratios, spanning from an excess of MAI with respect to CD to the opposite extreme (see experimental details in the ESI†). The reference system is pure MAI, which presents one singlet resonance at 7.46 ppm (NH_3_ group) and one at 2.36 ppm (CH_3_ group) in the ^1^H NMR spectrum (Fig. S2†). It is noteworthy that for the MAI/α-CD mixture the shape and sharpness of the NH_3_^+^ peak are severely compromised by the addition of the organic cavity, and the multiplicity of the –CH_3_ signal, starting from the molar ratio MAI/α-CD 1 : 4, drastically changes from singlet to quartet (Fig. 2a). This is probably due to the slower exchange processes of N–H protons as a consequence of the α-CD complexation. On the other hand, the addition of β-CD and γ-CD does not significantly alter the MA^+^ signals (Fig. 2a), thus suggesting weaker interactions. Further insight into the interaction/complexation mechanism of MAI-CDs is given by the study of the diffusion coefficient (*D*) correlated to the hydrodynamic radius (*r*_H_) of MAI in solution according to the Stokes–Einstein equation (eqn (1)), strictly holding for spherical molecules:^34^–^36^

1 *D* = *kT*/(6π*ηr*_H_)

where *k* is the Boltzmann constant, *T* the absolute temperature and *η* the dynamic viscosity of the solution. Taking into account the previously proven self-aggregating propensity of MAI,^7^ the interaction with CDs could lead to different and simultaneously occurring processes: (i) the inclusion into the hydrophobic CD cavity and/or interaction with the hydrophilic external surface of the macrocycles, which would cause an apparent increase in MAI molecular sizes and, hence, a decrease of its *D* value; (ii) the disaggregation of MAI aggregates due to the intercalation of CD molecules, thus causing a *r*_H_ reduction and a consequent increase in *D*. The mentioned processes can be identified by investigating the dependence of the MAI diffusion coefficient on the CD concentration, as shown in the collection of diffusion coefficients measured at different CD concentrations, shown in Fig. 2b–d and in Table S1.† The first interesting information we can extract is that the diffusion coefficient of MAI in the presence of β- or γ-CD is higher (10.1 × 10^–10^ m^2^ s^–1^ for β-CD and 9.3 × 10^–10^ m^2^ s^–1^ for γ-CD at a 1 : 0.028 molar ratio) than that of the free MAI (8.8 × 10^–10^ m^2^ s^–1^) until a molar ratio of 1 : 0.05 and 1 : 0.5 is reached for β- and γ-CD, respectively (Table S1†), thus indicating the propensity of β- and γ-CD to disfavor the MAI self-aggregation tendency, as expected with the occurrence of weak interactions mainly involving the external CD surface. In particular, β-CD induces the highest increase in the NMR parameter (*D* = 10.1 × 10^–10^ m^2^ s^–1^), withstanding a higher slope, as clearly highlighted in Fig. 2c. With an excess of β-CD and γ-CD, *D* decreases along with the increase in CD concentration (Fig. 2c–d and Table S1†), indicating that the complexation process becomes predominant in determining the measured diffusion coefficient. For α-CD, instead, *D* decreases even at very low CD concentrations and is always lower than that of pure MAI (Fig. 2b and Table S1†). This evidence clearly indicates the dominant inclusion process with α-CD, probably due to the better shape and size matching between the host cavity and guest. However, by increasing the amount of cyclodextrin, the contribution from the MAI disaggregation (leading to an increase in *D*) becomes dominant, likely due to the different complexation processes. The addition of CDs to MAI : PbI_2_ mixtures (Fig. S3 and Tables S2 and S3†) also shows a similar trend, demonstrating that α-CD strongly binds MA cations even in the presence of PbI_2_. Overall, the diffusion data show the higher propensity of β-CD to favor the disaggregation of MAI species, without strongly binding it. In contrast, the variation in the shape of the MAI signals (Fig. 2a) observed exclusively in the presence of α-CD, and the lower diffusion coefficient (Fig. 2b), suggests that the α-CD cavity strongly includes the perovskite organic moiety.

The differences in radii between the ions and internal cavities of the CDs probably account for the behavior of the present systems.^37^ Thus, MA^+^ (the ion radius is 1.8 Å)^38^ is preferentially lodged in the α-CD cavity (the internal cavity diameter of α-CD is 5.7 Å) rather than the bigger β-CD (7.8 Å) and γ-CD (9.4 Å) cavities (Fig. 1a). Taking into account the perovskite formation process, a strong inclusion of MAI in the CD cavity can likely be seen as a competing event to the MAI-PbI_2_ self-assembly, since it is supposed to impoverish the availability of MAI molecules for perovskite formation and for the coordination of PbI_2_ in solution. This, together with the lower intrinsic solubility of α-CD in the used solvents, justifies the negligible increase in the PbI_2_:MAI solubility with the addition of α-CD. On the other hand, the combination of the studies in solution and the complementary absorbance and NMR data clearly support the beneficial interaction selectively induced by the β-CD macrocycle with both the precursors, because of the optimal combination of cavity size and polarity. This leads, at the macroscopic level, to the improved PbI_2_ solubility based on a disaggregation of MAI and a simultaneous intercalation of β-CD between PbI_2_ layers that generates intermolecular spaces, large enough to accommodate a well-organized framework of organic components.

### From solution to solid state: photovoltaic devices and perovskite film formation assisted by β-CD

**Fig. 3:**
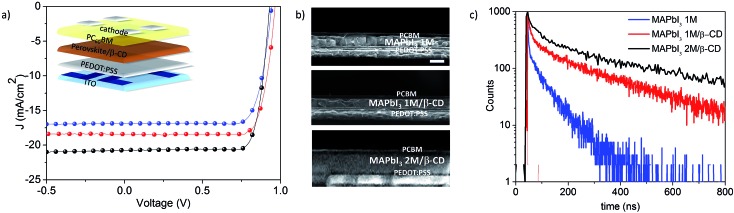
(a) Current–voltage curves for the best-performing devices; (b) cross section SEM images of PV devices. (c) Time-resolved photoluminescence decay curves using MAPbI_3_ 1 M (blue trace), MAPbI_3_ 1 M/β-CD (red trace) and MAPbI_3_ 2 M/β-CD (black trace) films.

Following the β-CD effect on the solution chemistry of perovskite precursors, we now study the next step towards the film formation. We characterize the properties of the film and verify whether the perovskite material, in the presence of the molecule, remains suitable, or becomes more suitable, for application in optoelectronic devices. As a proof of concept, we test our material in planar solar cells with inverted geometry comprising indium tin oxide (ITO)/PEDOT:PSS/MAPbI_3_/[6,6]-phenyl-C_60_-butyric acid methyl ester (PC_60_BM)/C_60_ (20 nm)/bathocuproine (BCP) (5 nm)/Al (a sketch of the device is shown in Fig. 3a). All details relating to the device optimization and characterization, statistics, CD comparison and concentration screening are reported in the ESI – Section 2 (Fig. S4 and S5†). We compare the films deposited from 1 M solutions, with and without β-CD, namely MAPbI_3_ 1 M and MAPbI_3_ 1 M/β-CD. Importantly, we found that the presence of the organic cavity inside the perovskite layer, in a concentration of 3.6 wt% as quantified by the NMR spectrum of the dissolved film (Fig. S6†), is not detrimental to the device performance, but rather allows a boost in the power conversion efficiency (PCE) from 12.4% to 14.1%. Taking advantage of the improved performances, we test the double concentrated solution achieved in the presence of β-CD, namely MAPbI_3_ 2 M/β-CD, in the photovoltaic device and the current–voltage (*J*–*V*) curves are shown in Fig. 3a. It is noteworthy that the active layer thickness almost doubled due to the use of a more concentrated solution, as shown in the scanning electron microscopy (SEM) cross sections in Fig. 3b, allowing an increase in the short circuit current (*J*_sc_) from 16.4 mA cm^–2^ to 21 mA cm^–2^. We additionally observed a very high fill factor (FF) for the MAPbI_3_ 2 M/β-CD cell (0.81 *vs.* 0.79 of the reference), overall leading to an enhanced PCE of 16%.^39^–^41^ It is worth noting that the device shows no hysteresis (Fig. S4a†) and a perfectly stabilized maximum power point over time (Fig. S4c†). To gain further confirmation of the negligible effect of CD molecules on the electrical properties of the film, besides the high FF of 81%, we measured the series resistance (*R*_s_) for the devices embedded or not embedded with β-CD. In particular, the reciprocal *R*_s_, calculated as the slope at the open-circuit voltage (*V* = *V*_OC_) of the illuminated *J*–*V* curves, was plotted against the short-circuit current for different light intensities (Fig. S7†). As previously demonstrated,^42^,^43^ the difference in *R*_s_ can be mainly ascribed to the intrinsic charge transport properties of the active layer. Fig. S7† shows the 1/*R*_s_ trend and provides evidence of a similar slope for the MAPbI_3_ 1 M and MAPbI_3_ 2 M/β-CD devices, confirming that the charge transport properties are substantially unchanged despite the inclusion of β-CD.

The optical properties of the perovskite films are monitored using steady-state (Fig. S8†) and time-resolved photoluminescence (PL) measurements (Fig. 3c). We tested our films under low excitation fluence and at room temperature, conditions in which relatively low PL quantum yield (PLQY) values are expected due to the predominant trap recombination deactivation mechanism impoverishing the reduced excited state populations.^44^ An increasing difference is found for MAPbI_3_/β-CD films in comparison to pristine perovskite (PLQY of 0.8% for MAPbI_3_ 2 M/β-CD, 0.4% for MAPbI_3_ 1 M/β-CD and 0.3% for MAPbI_3_ 1 M). In particular, time-correlated single-photon counting measurements give information on the PL quenching sites. As the MAPbI_3_ film contains β-CD, the average deactivation paths (*τ*) gradually increase from *τ*_1_ = 4 ns and *τ*_2_ = 67 ns for MAPbI_3_ 1 M to *τ*_1_ = 13 ns and *τ*_2_ = 230 ns for MAPbI_3_ 1 M/β-CD films to *τ*_1_ = 71 ns and *τ*_2_ = 600 ns for MAPbI_3_ 2 M/β-CD. Clearly, the presence of β-CD has a beneficial effect on the carrier recombination mechanisms occurring within the films, even at a 1 M concentration.

**Fig. 4:**
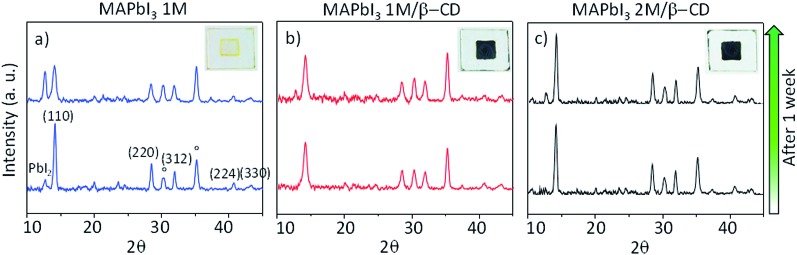
Thin-film theta/2theta XRD patterns of (a) pristine MAPbI_3_ 1 M, (b) MAPbI_3_ 1 M/β-CD and (c) MAPbI_3_ 2 M/β-CD perovskite films deposited on ITO (°) substrates which were exposed to ambient air, before and after degradation. Inset: pictures of corresponding films after 3 months in air.

**Fig. 5:**
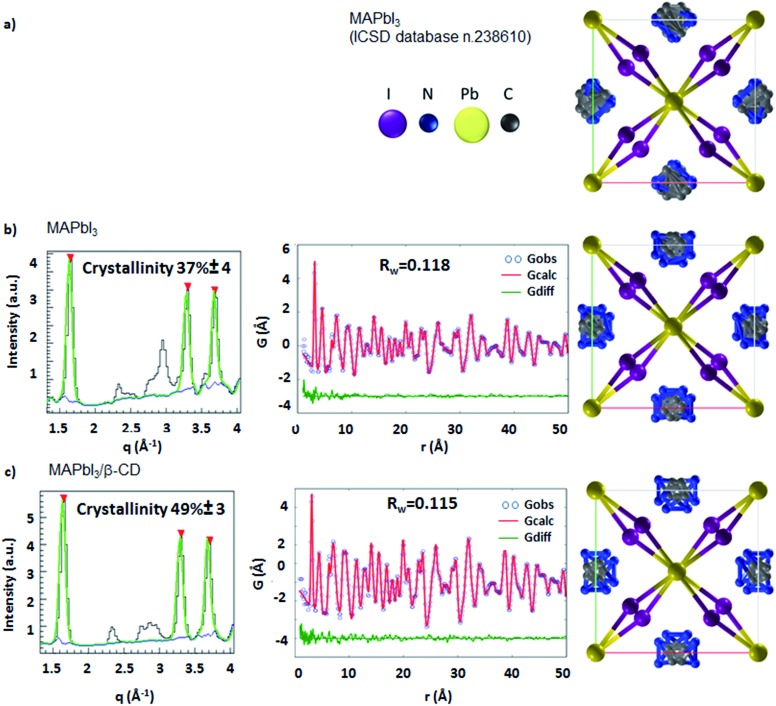
From left to right: determinations of crystallinity using synchrotron X-ray powder diffraction profiles (black lines); PDF fits with weighted profile agreement factors (*R*_w_); tetragonal MAPbI_3_ perovskite structures as seen along the *c* axis. The highest peaks are fitted by Gaussian functions (green line) added to the background (blue curve) estimated by the SNIP algorithm (reference in the main text). The crystallinity is measured as the ratio between the integral of the Gaussian curves with respect to the integral of the Gaussian + background curves. The percentage of crystalline to amorphous signal is calculated through a XPD fitting procedure from: (a) ICSD database n. 238610 in the space group *I*4/*mcm*; (b) resulting from the fitted PDF data for the pristine sample and (c) for the sample with β-CD.

**Fig. 6:**
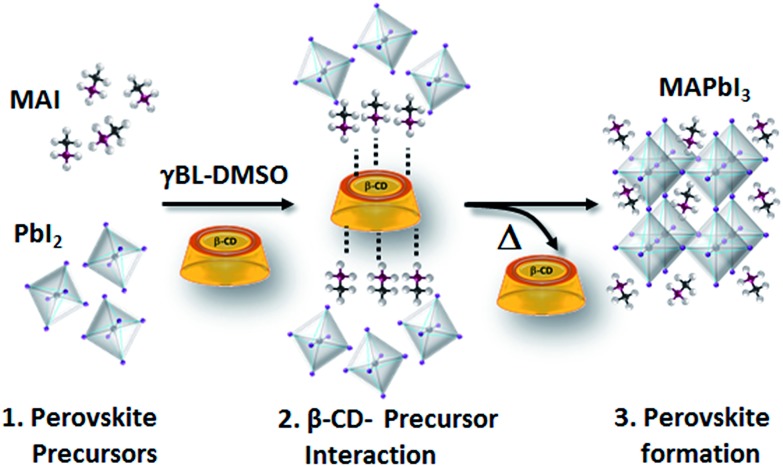
Simplified sketch of the possible β-CD working mechanism on perovskite formation.

Morphological characterization of the three films is shown in Fig. S9.† SEM and atomic force microscopy (AFM) images show more compact and merged grains for the β-CD/perovskite material with both the 1 M and 2 M concentrations. Importantly, we verify that the presence of CD, in addition, results in an improved resistance to decomposition of the perovskite material under ambient conditions. This is clearly shown in Fig. 4, a collection of XRD spectra of perovskite films recorded at defined times of continuous exposure to the environment (ambient air ≈ 70% relative humidity, room temperature = 25 ± 1 °C)^45^,^46^ for 170 hours. We find that the pristine perovskite (Fig. 4a) is severely corroded by moisture, showing a clear increment of the PbI_2_ peak at 12.6° at almost the same intensity as the MAPbI_3_ main reflection at 14.1°, meanwhile β-CD containing films are stable under these conditions, maintaining the original XRD patterns. The formation of a more compact film can possibly contribute to slowing down the permeation of moisture and oxygen through the bulk. To further rationalize the improved moisture stability, high PL and good device performance, and to gain deep insight into the role played by β-CD in the formation of the perovskite structure, MAPbI_3_ powders with and without the inclusion of β-CD are investigated using synchrotron X-ray powder diffraction (XPD)/pair distribution function (PDF). A qualitative phase analysis of the XRD patterns from powders (Fig. S10†) indicates an unaltered perovskite tetragonal structure, independent of the presence of β-CD. Furthermore, no additional structural signal ascribable to CD’s possible incorporation in the lattice is found, indicating that the CDs are located outside the crystalline lattice, possibly in the amorphous phase or at the grain boundaries.^44^ This is the first important proof of the process of CD exclusion from the perovskite framework during the film deposition/formation. Analysis of the atomic pair distribution function (PDF) calculated from the synchrotron measurements is also carried out; this allows a higher sensitivity to light atoms and to non-crystalline components. Fig. 5b and c also include an estimate of the crystalline phase amount calculated from a profile fitting procedure applied to XRD patterns. A significant relative increase in perovskite crystallinity is found in the β-CD embedding film, 49%, with respect to 37% for bare MAPbI_3_ (inset Fig. 5b and c). The crystalline moiety data can be fitted with good agreement using a linear superposition of tetragonal MAPbI_3_ and the PbI_2_–MAI–DMSO intermediate phase (relative concentrations in Table S7†). The formation of this intermediate crystalline phase is well documented for perovskite films prepared from DMSO solutions^47^ and is ascribed to the Pb-coordinating ability of this solvent. No distances resulting from the PDF can be attributed to the CD atomic structure, in agreement with the XPD measurements described above. This confirms that β-CD is present in a small amount, as seen by NMR spectroscopy (Fig. S6†), and with no periodic organization. Small scale deviations between the data and fit model (see the PDF difference profiles in Fig. 5) can be ascribed to the presence of nanocomponents with a diameter of < 2 nm or to second order inaccuracies in the model parametrization (for example atomic scattering factors have been used instead of ionic ones). Notably, analysis of the structural models, refined by PDF data, suggests further interesting features: (i) slightly different values are found for the tilting angles between successive octahedra in the *a*–*b* plane. Such a feature is quantified by measuring the torsion angle I–Pb–Pb–I^48^ along the *c* axis (see Fig. S11 and the values reported in Table S7†), which is found to be larger for MAPbI_3_/β-CD; (ii) the difference between the C–I and N–I average distances increases in perovskites/CD (Fig. S12†). In terms of the MA position in the cage, it appears that the NH_3_^+^ features favorably point toward the iodide, allowing the stronger NH_3_^+^–I^–^ interactions to prevail over the weaker CH_3_–I^–^ interactions^48^ in the MAPbI_3_/β-CD sample. This preferential orientation likely contributes to stabilizing the MAPbI_3_/CD material. It should be noted, as a further confirmation of the validity of our observations, that similar results can be obtained using an alternative MAPbI_3_ structural model with *I*4*cm* instead of *I*4/*mcm* symmetry. In summary, the improved crystallinity with respect to bare MAPbI_3_ and the preferential MA orientation could both contribute to the stabilization of the MAPbI_3_/β-CD composite towards moisture degradation. In fact, if a more compact film would reduce the permeation of moisture and oxygen through the bulk, the higher degree of crystallinity would make the material itself more resistant to external agents and decomposition. The PDF measurements also suggest a further beneficial effect of β-CD on the carrier recombination process within the films; in fact, the CD molecules could act as grain surface passivation agents, given their preferential location outside the crystallites, reducing non-radiative trap mediated recombination.^49^ The collection of the advanced structural investigations on MAPbI_3_ and MAPbI_3_/β-CD systems also allows depiction of the mechanism of action of the organic cavity on the perovskite formation. The results demonstrate that β-CD does not interfere with the unit cell structure of perovskite being excluded from the crystalline phase during the evaporation of the solvent and only interacts with the solvated ionic species in solution, acting in this system as a promoter of the reaction between MAI and PbI_2_, positively affecting the properties of the final film. A representative sketch of the proposed mechanism is shown in Fig. 6. The role played by CD is likely induced by the unique supramolecular network established in solution that affects the mechanism of perovskite formation.

## Conclusions

Herein, we introduce the concept of using traditional molecular hosts, cyclodextrins, to generate a hybrid perovskite-soft material. We demonstrate that the interaction between CDs and perovskite precursors, MAI and PbI_2_, leads to the formation of a supramolecular organic–inorganic hybrid framework importantly modifying the solution chemistry and the final film properties. In particular, our study including α-, β- and γ-CDs identifies β-CD as the best compromise between cavity size and hydrophilicity, inducing cooperative MA^+^/β-CD and PbI_2_/β-CD interactions that, overall, allow the equilibria of the perovskite precursors and their solubility limit in the reaction media to be significantly overcome. This leads to more concentrated solutions, thus to thicker active layer films. We shed light on the mechanism involving β-CD and the role it plays both in solution and in films. The supramolecular interactions established by β-CD, as well as its preferential location in the film at the grain boundaries, in fact, lead to very important consequences for the final polycrystalline film: (i) improving the crystalline/amorphous ratio; (ii) inducing a stronger interaction between the MA^+^ cation and I^–^ in the lattice; (iii) enhancing the optoelectronic properties of the active layer for an ultimate decrease in carrier deactivation; (iv) enhancing the moisture stability of the resulting polycrystalline film. All of this leaves the suitability of the material to be employed in optoelectronic devices untouched. It is noteworthy that the exploitation of a few additives aimed at the improvement of perovskite film properties has been reported, but the use of CDs provides the unique two-fold advantage of a complexation with the MA^+^ cation concurrent with an out of cage PbI_2_ intercalation. This is of paramount importance as it links the complex equilibria in solution of perovskite precursors with positive repercussions in their self-assembly process. Our results introduce a new class of promoter for perovskite formation, which presents a great potential for a wide range of device-related applications, as well as for the development of tailored composite materials.

## Conflicts of interest

There are no conflicts to declare.

## Supplementary Material

Supplementary information
