# Supplementary material for: Connecting the solution chemistry of PbI2 and MAI: a cyclodextrin-based supramolecular approach to the formation of hybrid halide perovskites
Source: Chem Sci. 2018 Feb 12;9(12):3200–8. doi: 10.1039/c7sc05095j (PMC5916222; doi:10.1039/c7sc05095j)
Supplement: Supplementary file 1 [file SC-009-C7SC05095J-s001.pdf]

Electronic Supplementary Material (ESI) for Chemical Science.
This journal is © The Royal Society of Chemistry 2018

Supporting Information

Connecting the solution chemistry of PbI_2 and MAI: a cyclodextrin-based supramolecular approach to the formation of hybrid halide perovskites

Sofia Masi,[a,b] Federica Aiello,[c] Andrea Listorti,[a,b] Federica Balzano,[c] Davide Altamura,[d] Cinzia Giannini,[d] Rocco Caliandro,[d] Gloria Uccello-Barretta,[c] Aurora Rizzo[a*] and Silvia Colella[a,b*]

a Istituto di Nanotecnologia CNR-Nanotec, DistrettoTecnologico via Arnesano 16, 73100 Lecce, Italy

b Dipartimento di Matematica e Fisica "E. De Giorgi", Università del Salento, Via per Arnesano, 73100 Lecce, Italy

c Dipartimento di Chimica e Chimica Industriale, Università di Pisa, Via Moruzzi 13, 56124 Pisa, Italy

d Istituto di Cristallografia, CNR-IC, Via Amendola 122/O, 70126 Bari, Italy

Contents

Experimental section 1
Section 1. Solution characterization 3
Section 2. Solar cells optimization and characterization 5
Section 3. Thin film characterization 8
Section 4. Advanced structural characterization 10
References 11

Experimental section

Materials. All materials were purchased from Sigma-Aldrich or Alfa Aesar and used as received. CH_3NH_3I was synthesized according to a reported procedure.[1] CH_3NH_2 (27.86 ml, 40% in methanol, TCI) and hydroiodic acid (30 ml, 57 wt% in water, Aldrich) were mixed at 0 °C and stirred for 2 h. The precipitate was recovered by evaporation at 50 °C for 1 h. The product was washed with diethyl ether three times and finally dried at 60 °C in a vacuum oven for 24 h. Deuterated dimethylsulfoxide (DMSO-d6) was purchased from Deutero GmbH and employed without further purification.

SEM. The SEM imaging was performed by the MERLIN Zeiss SEM FEG instrument at an accelerating voltage of 5 kV, using an In-lens detector.

AFM imaging was carried out in air using a Park Scanning Probe Microscope (PSIA) operating in a noncontact mode to reduce tip induced surface degradation and sample damage.

XRD. The XRD spectra of the prepared films were measured with a PAN alyticalX'Pert-PRO Materials Research Diffractometer using graphite-monochromated CuKα radiation (λ = 1.5405 Å).

Synchrotron XRD/PDF. X-ray synchrotron data were collected at the 28-ID-2beamline of the National Synchrotron Light Source (NSLS-II) at Brookhaven National Laboratory (USA). They were measured with a X-ray energy of 67.5 keV (0.18372 Å). A large area 2D Perkin Elmer detector (2048x2048 pixels and 200x200 μm pixel size) was mounted orthogonal to the beam path, 348 mm downstream the sample. The detector position was chosen so as to collect data over a large Q-range ($Q = (4\pi/\lambda)\sin\theta$, scattering vector modulus) data suitable for PDF analysis, but with still good angular resolution for XPD analysis. LaB_6 was measured as the standard material to calibrate the wavelength and the sample-to-detector distance, and to unfold raw 2D data into azimuthally integrated intensity versus Q1D profiles, by using the program FIT2D.[2] Single measurements were carried out on powder samples by using glass capillaries of 1 mm diameter. An empty capillary was measured for background estimation. Powder samples were obtained by scraping the perovskite films from the substrate.

Synchrotron data analysis. Crystallinity fraction was estimated from XPD profiles by using the program RootProf.è.[3] The signal from the highest peaks was fitted by a sum of Gaussians, while background was estimated by the SNIP clipping algorithm.[4] PDF profiles were calculated by the program PDFGetX3 [5] in the Q-range from to 0.1 $Å^{-1}$ to 22 $Å^{-1}$. The crystal structures identified on the basis of the preliminary screening using XPD profiles were fitted against PDF profiles with the program PDFgui.[6] Each sample was best fitted by using a model constituted by a linear superposition of the tetragonal $MAPbI_3$ crystal structure (ICSD database n.238610)[7] and the orthorhombic PbI_2-CH_3NH_3I-DMSO crystal structure.[8] Similar results were also obtained by using an alternative tetragonal $MAPbI_3$ crystal structure (COD database n.4335638),[9] which has a lower symmetry space group (I 4 c m instead of I 4/m c m). Hydrogen atoms have been removed from the original structural model. Cell parameters, atomic positions and thermal factors of the $MAPbI_3$ crystal phase, and only cell parameters of the PbI_2-CH_3NH_3I-DMSO crystal phase were varied during the fit. Crystal structures were displayed by the viewer JAV.[10]

PL. Steady state and time resolved photoluminescence was measured by an Edinburgh FLS920 spectrometer equipped with a Peltier-cooled Hamamatsu R928 photomultiplier tube (185–850 nm). An Edinburgh Xe900 450 W Xenon arc lamp was used as exciting light source. Corrected spectra were obtained via a calibration curve supplied with the instrument. (lamp power in the QY experiments ~ 0.6 $mWcm^{-2}$, spot area 0.5 cm^2) Emission lifetimes were determined with the single photon counting technique by means of the same Edinburgh FLS980 spectrometer using a laser diode as excitation source (1 MHz, λexc = 635 nm, 67 ps pulse width and about 30 ps time resolution after deconvolution) and a Hamamatsu MCP R3809U-50 (time resolution 20 ps) as detector. (laser power in the TRPL experiment ~ 1.6 [illegible]Wcm^{-2}, spot area 0.3 mm^2) Perovskite thin film sealed with a 50-nm-thick poly(methylmethacrylate) (PMMA) layer to avoid degradation under air exposure.

NMR. NMR measurements were performed on a Varian Inova spectrometer operating at 600 MHz for ^1H nuclei; the temperature was controlled to ± 0.1°C. DOSY (Diffusion Ordered SpectroscopY) experiments were carried out by using a stimulated echo sequence with self-compensating gradient schemes and 64K data points. Typically, g was varied in 20 steps (16 transients each) and Δ and δ were optimized in order to obtain an approximately 90-95% decrease in the resonance intensity at the largest gradient amplitude. The baselines of all arrayed spectra were corrected prior to processing the data. After data acquisition, each FID was apodized with 1.0 Hz line broadening and Fourier transformed. The data were processed with the DOSY macro (involving the determination of the resonance heights of all the signals above a pre-established threshold and the fitting of the decay curve for each resonance to a Gaussian function) to obtain pseudo two-dimensional spectra with NMR chemical shifts along one axis and calculated diffusion coefficients along the other. Gradient amplitudes in DOSY experiments have been calibrated by using a standard sample of D_2O 99%. TMS has been used as internal standard for viscosity. Its diffusion coefficient (7.32 x 10^{-10} m^2/s) and longitudinal relaxation time (6.83 s) remained unchanged in the presence and in the absence of cyclodextrins. Binary mixtures MAI/CD or $MAPbI_3$/CD were prepared in DMSO-d6 by employing a 5 mM concentration for MAI or $MAPbI_3$, and with the following molar ratios: 1:0.028, 1:0.05, 1:0.1, 1:0.5, 1:1, 1:2, 1:4, 1:6, 1:8, 1:10. For binary mixtures prepared with β-CD and γ-CD, the samples were analyzed after 20 h from preparation, whereas for binary mixtures prepared with α-CD, the mixtures MAI/CD and $MAPbI_3$/CD were analyzed after 21 days from preparation; the mixtures MAI/CD added by PbI_2 were left to equilibrate for 2 days before the analysis.

Device fabrication and characterization. ITO-coated glass substrates 4L etched (Visiontech) were cleaned by ultrasonication in deionized water, 2-propanol and acetone. Substrates were treated to the TL1-washing procedure (washed in double distilled water (Milli-Q water), hydrogen-peroxide (H_2O_2) and ammonia (NH_3) 5:1:1 v/v (at 80°C for 10 minutes) to remove organic contamination, then rinsed ten times in water prior next depositions. A 40 nm-thick PEDOT:PSS layer was then deposited on the substrates by spin coating at 3,000 r.p.m. for 60 sec and annealed at 140 °C for 20 min. The prepared CH_3NH_3I and commercial PbI_2 (ultra dry, 99.999% (metals basis) Alfa Aesar) for the 1M $CH_3NH_3PbI_3$ solution were stirred in a mixture of γ-butyrolactone (GBL) and DMSO (2:1 vol/vol; GBL, ≥99%; DMSO, 99.8%; Sigma-Aldrich) at 60 °C for 12 h. The 2M perovskite precursor solution containing cyclodextrin was prepared as follows. CH_3NH_3I (2 M), PbI_2 (2 M) and 1, 2, 5 wt% of α– β- or γ-CD were dissolved in 1,5 ml GBL/DMSO (2:1vol/vol) mixed solvent with stirring overnight at 60 °C and then stored in a dry box (humidity <1%) at room temperature. Before use, these precursor solutions were filtrated using a hydrophilic PTFE syringe filter (pore size of 22 μm). The filtrated perovskite precursor solution was coated onto PEDOT:PSS-ITO substrate by a consecutive two-step spin-coating process at 1,000 and 4,000 r.p.m. for 10 and 60 s, respectively with a dipping of dicloromethane (DCM) at 10 sec to the end. The optimized devices have a precursor ratio of $MAPbI_3$ 2M/β-CD 5 wt%, thickness of 250 nm. Its thermal annealing time of perovskite film was optimized to 10 minutes under 100 ℃. After cooling to room temperature, the $PC_{60}BM$ solution was spin-coated on the perovskite layer at 1,000 r.p.m. for 60s. A $PC_{60}BM$ solution was prepared by dissolving 25 mg in 1 ml chlorobenzene (99.8%, Sigma Aldrich). This fabrication process was carried out under controlled atmospheric conditions with a humidity of <1% and a temperature between 20 and 25 °C. Finally, 0.5 nm of LiF or 20 nm of C_{60} and 5 nm of BCP and 150 nm of aluminum were thermally evaporated on top of the device at a pressure of 5 × 10^{-6} mbar for 30 min to form the back contact. The devices were characterized using a Keithley 2400 Source Measure Unit and AirMass 1.5 Global (AM 1.5G) solar simulator (Newport 91160A) with an irradiation intensity of 100 mW/cm^2. The solar simulator irradiance was set to 100 mW/cm^2 using a thermopile radiant power meter with fused-silica window (Spectra

Physics Oriel, model 70260). The active area of the complete device was 0.04 cm^{-2}. All devices are tested using 100 mV/s or 1000 mV/s scan rates, under nitrogen at 22 °C.

The IPCE was measured using a power source (Newport 300W xenon lamp, 66920) with a monochromator (Newport Cornerstone 260), a power meter (Newport 2936-C) a photodiode (Newport 71675_71580) and a multimeter (Keithley 2001). The characterization is performed in air on encapsulated devices.

UV-Vis absorption. Ultraviolet-visible absorption spectra were recorded on Varian Cary 500 spectrophotometer in the 350–1,000 nm wavelength range at room temperature. The perovskite precursor solution containing cyclodextrin was prepared as follows. A diluted solution of PbI_2 (1 wt%) with an increasing amount of α- β- or γ-CD (molar ration 1:10, 1:20, 1:30, 1:40) were dissolved in 1,5 ml GBL/DMSO (2:1 vol/vol) mixed solvent with stirring overnight at 60 °C. The solubility of perovskite precursors was determined from the concentration of saturated solutions.

Section 1. Solution characterization

Rheological analysis. Steady shear viscosities (η) of the perovskite materials were determined using an Anton Paar Phisica MCR 301 instrument equipped with measuring cone plate geometry (CP25-1 with 24.980 mm diameter and angle 1°). First, dynamic frequency spectra were conducted in the linear viscoelastic regime of the samples, as determined from dynamic stress sweep measurements. Dynamic stress sweeps were conducted at a constant frequency of 1 Hz. The steady shear viscosity was measured as a function of the shear rate in a range of 0.01–1000 s^{-1}.

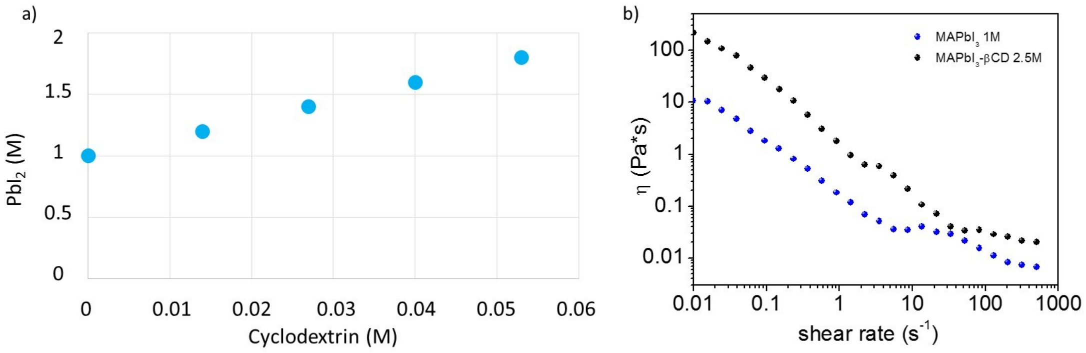

Figure S1. a) Solubility diagram of PbI_2 in GBL:DMSO 2:1 solvent mixture under stirring at 70°C. b) Comparison of the shear viscosity for $MAPbI_3$ 1M and $MAPbI_3$ 2.5M/β–CD solutions.

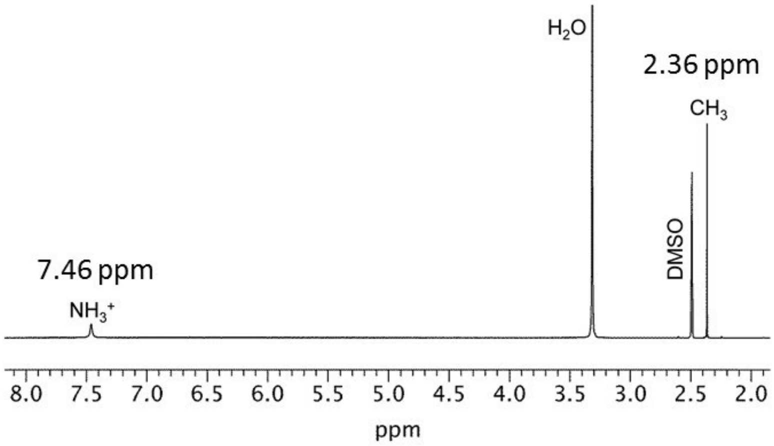

Figure S2. ^1H NMR spectrum (600 MHz, DMSO-d6, 25 °C) of MAI (5 mM).

Table S1. Diffusion coefficient (D, x10^{10} m^2/s) of MAI (5mM) in the presence of increasing amounts of CDs.

Molar ratio MAI/CD	α–CD	β–CD	γ–CD
1:0	8.8	8.8	8.8
1:0.028	8.3	10.1	9.3
1:0.05	8.2	10.3	9.5
1:0.1	7.9	9.9	9.5
1:0.5	6.2	9.6	9.6
1:1	5.9	8.4	9.2
1:2	5.1	8.1	8.9
1:4	6.2	7.7	8.8
1:6	6.5	7.1	8.7
1:8	6.7	7.0	8.4
1:10	6.9	7.0	7.7

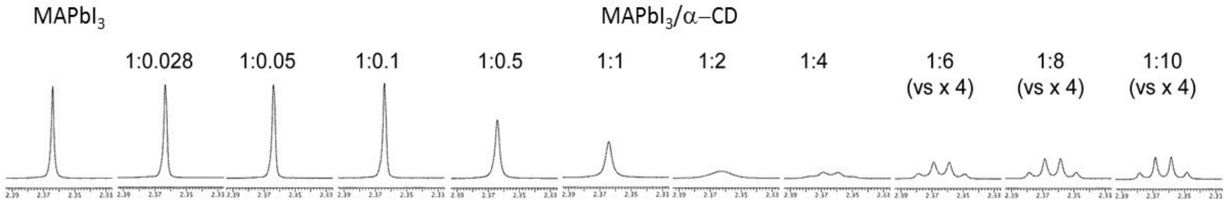

Figure S3. ^{1}H NMR (600 MHz, DMSO-d6, 25 °C) expansions of CH_3 signal of free MAI+PbI_2 (5 mM) and mixtures MAI+PbI_2/α-CD at different molar ratios.

Table S2. Diffusion coefficient (D, x10^{10} m^2/s) of MAI (5mM) in the presence of increasing amounts of CDs with the addition of equimolar amounts PbI_2. Data acquired by adding PbI2 to the mixtures MAI/CD.

Molar ratio MAI/CD	α–CD	β–CD	γ–CD
1:0	8.9	8.9	8.9
1:0.028	7.9	9.3	9.2
1:0.05	8.2	9.6	9.2
1:0.1	8.0	9.1	9.2
1:0.5	8.0	9.0	9.2
1:1	6.5	8.6	9.1
1:2	5.1	8.8	9.0
1:4	4.6	8.6	8.7
1:6	4.6	7.9	8.9
1:8	5.2	8.2	8.1
1:10	5.6	7.2	7.5

Table S3. Diffusion coefficient (D, x10^{10} m^2/s) of MAI+PbI_2 previously mixed (5mM), with the addition of increasing amounts of CDs.

Molar ratio $MAPbI_3$/CD	α–CD	β–CD	γ–CD
1:0	8.3	8.3	8.3
1:0.028	8.2	9.2	9.3
1:0.05	7.9	9.6	9.3
1:0.1	7.6	9.6	9.5
1:0.5	7.5	9.3	9.8
1:1	7.6	9.2	9.2
1:2	5.7	8.5	8.8
1:4	5.8	8.0	9.1
1:6	6.9	7.9	8.4
1:8	7.3	7.4	7.8
1:10	6.6	7.3	7.9

Section 2. Solar cells optimization and characterization

The β-CD/perovskite film was obtained through a previously optimized spin-coating process using dichloromethane (DCM) as anti-solvent.[11,12]

The whole optimization of PV devices with and without CDs inclusion has been performed on the semplified architecture ITO/PEDOT:PSS/perovskite/PCBM/LiF/Al. Results related to the different CDs at constant concentration of 2wt% in a 1M $MAPbI_3$ solution are reported, highlighting how incresing the β-CD concentration up to 5 wt% (according tot he solubility curve in Figure 1) allowed to achieve a 2M perovskite precursor concentration, leading to the best performing device showing V_{OC} 0.96 V, J_{SC} 18.4 mA/cm^2, FF 82% and PCE 14.6%. The screening of β-CD concentration both for 1M and 2M is reported in Table S5.

Carrying on a further optimization of the device layout, by adding a C_{60} (20 nm) and a hole blocking Bathocuproine (BCP) (5nm) thin interlayers before Al cathode, we reach a PCE =16% for the $MAPbI_3$ 2M/β-CD, a PCE = 14.% for $MAPbI_3$ 1M/β–CD and a PCE = 12.4% for $MAPbI_3$ 1M, reported in Figure 3 (main text). The direct comparison between the two cathods are reported in Table S6. Incident photon-to-current conversion efficiency (IPCE) and the curves in direct and forward sweep directions are also shown in Figure S4, highlighting no hysteresis.The statistic distribution for both architectures are shown in Figure S5.

Table S4. Solar cell figures of merit devices ITO/PEDOT:PSS/$MAPbI_3$/$PC_{60}BM$/LiF-Al fabricated with and without CDs at 2 wt% and 5 wt%. Open-circuit voltage (V_{OC}), short-circuit current density (J_{SC}), fill factor (FF), and photocurrent efficiency (PCE).

CD	**$MAPbI_3$ 1M, CDs 2 wt%**				**$MAPbI_3$ 2M- CD 5 wt%**			
	PCE (%)	FF	Voc (V)	Jsc (mA/cm^2)	PCE (%)	FF	Voc (V)	Jsc (mA/cm^2)
w/o CD	9.3	0.76	0.93	13.1	/	/	/	/
α-CD	6.4	0.68	0.86	10.3	/	/	/	/
β-CD	9.9	0.80	0.88	14.1	14.6	0.82	0.96	18.4
γ-CD	6.7	0.79	0.87	9.4	/	/	/	/

Table S5. Solar cell figures of merit devices ITO/PEDOT:PSS/$MAPbI_3$/$PC_{60}BM$/LiF-Al fabricated with increasing amount of β–cyclodextrin.

**$MAPbI_3$ 1M**					**$MAPbI_3$ 2M**			
β-CD wt (%)	PCE (%)	FF	Voc (V)	Jsc (mA/cm^2)	PCE (%)	FF	Voc (V)	Jsc (mA/cm^2)
0%	9.30	0.76	0.93	13.10	/	/	/	/
1%	9.50	0.80	0.94	12.65	/	/	/	/
2%	9.90	0.80	0.88	14.10	/	/	/	/
5%	8.23	0.70	0.97	12.07	14.63	0.82	0.96	18.40
10%	7.21	0.78	0.90	10.23	11.46	0.68	0.94	17.80

Table S6. Solar cell figures of merit devices fabricated with and without β-cyclodextrins, with architectures ITO/PEDOT:PSS/$MAPbI_3$/$PC_{60}BM$/LiF-Al and ITO/PEDOT:PSS/$MAPbI_3$/$PC_{60}BM$/C_{60}-BCP-Al.

	cathode	PCE (%)	FF	V_{OC} (V)	J_{SC} (mA/cm^2)
$MAPbI_3$ 2M/β-CD	LiF/Al	14.6	0.82	0.96	18.4
$MAPbI_3$ 2M/β-CD	C_{60}/BCP/Al	16.0	0.81	0.94	21.0
$MAPbI_3$ 1M/β-CD	LiF/Al	9.90	0.80	0.88	14.10
$MAPbI_3$ 1M/β-CD	C_{60}/BCP/Al	14.1	0.81	0.96	18.1
$MAPbI_3$ 1M	LiF/Al	9.3	0.76	0.93	13.1
$MAPbI_3$ 1M	C_{60}/BCP/Al	12.4	0.79	0.94	16.8

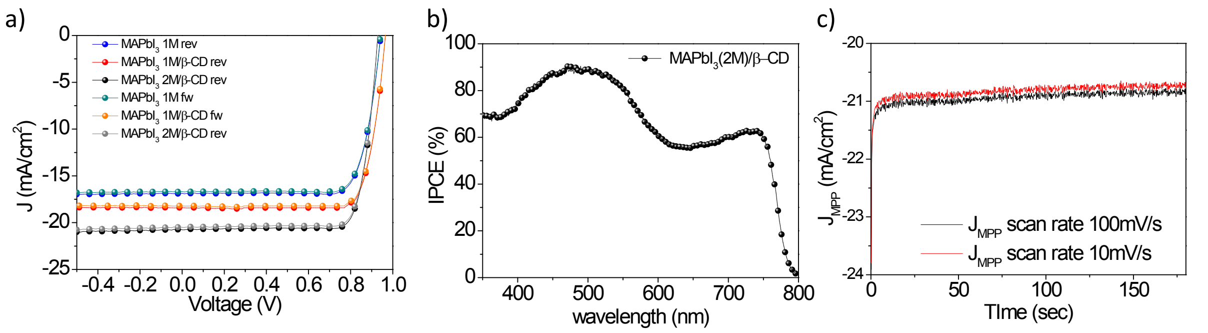

Figure S4. a) Current–voltage curves for the best-performing devices (architecture: ITO/PEDOT:PSS/$MAPbI_3$/$PC_{60}BM$/C_{60}-BCP-Al) using pristine (blue) and $MAPbI_3$ 2M/β-CD (black) perovskites under AM 1.5 at one sun in order to evidence no hysteresis for curve recorded in reverse scan (rev, from 1.0 V to -0.5 V) and curve recorded in forward scan (fw, from -0.5 V to 1.0 V) and b) the IPCE spectra of $MAPbI_3$ 2M/β-CD best performing device; the integrated currents from the product of the incident photon-to-electron conversion efficiency and the AM 1.5 100mW/cm^2 solar spectrum is 19.21 mA/cm^2 for $MAPbI_3$ 2M/β-CD, in close agreement to the short-circuit photocurrent measured under simulated sunlight. c) Steady-state current measured at a maximum power point (0.80 V) and stabilized power output for $MAPbI_3$ 2M/β-CD-based device.

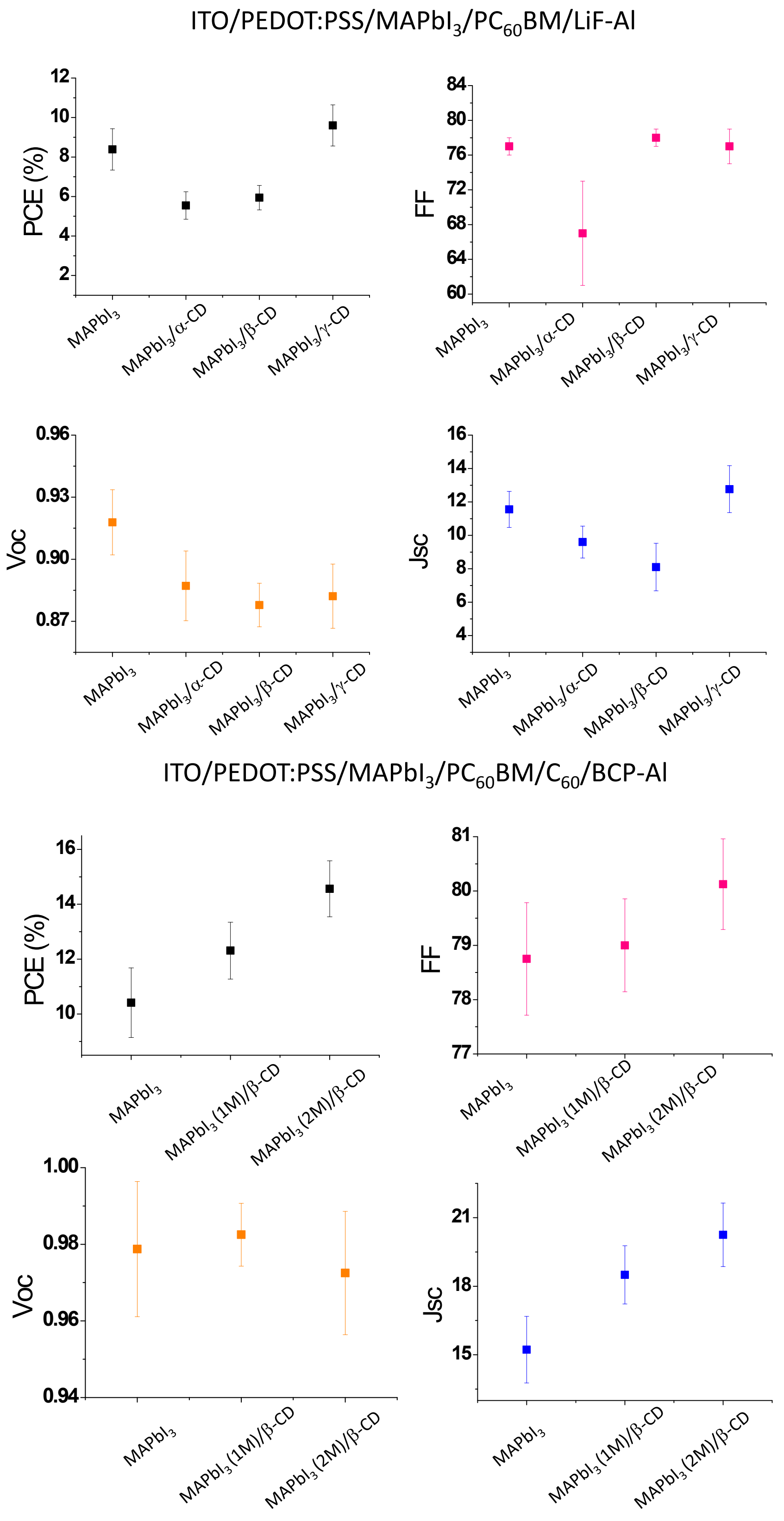

Figure S5. Efficiency values showed as plots for all relevant parameters distributions of the devices in ITO/ PEDOT:PSS/ $MAPbI_3$ 1M/ $PC_{60}BM$/LiF-Al configuration fabricated with and without cyclodextrins, and ITO/ PEDOT:PSS/$MAPbI_3$/ $PC_{60}BM$/C_{60}/BCP-Al configuration fabricated with $MAPbI_3$ 1M, $MAPbI_3$ 1M/β-CD and $MAPbI_3$ 2M/β-CD. Error bars represent the standard deviations and filled squares indicate mean values. Each value represents the distributions of 30 cells prepared under the same conditions.

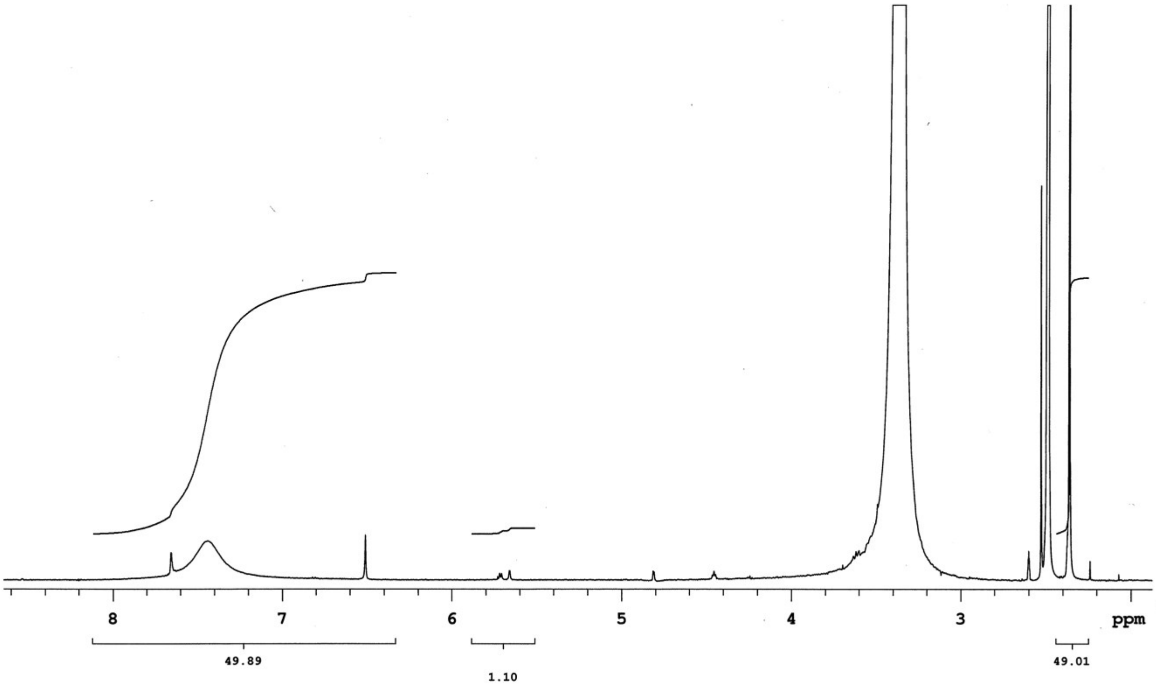

Figure S6. ^{1}H NMR spectrum (600 MHz, DMSO-d6, 25 °C) of dissolved $MAPbI_3$ 2M/β-CD film in DMSO-d6.

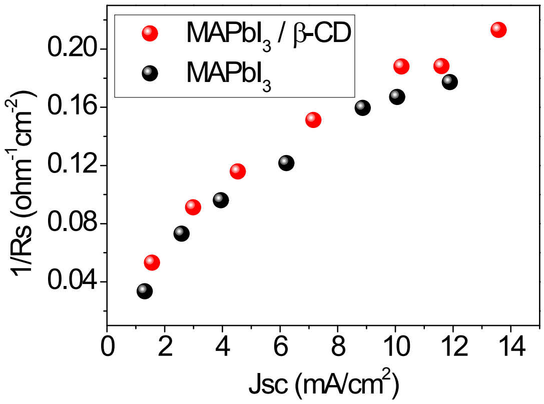

Figure S7. The relationship between reciprocal series resistance and short circuit current density (Jsc) for the cells fabricated from $MAPbI_3$ 1M and $MAPbI_3$ 2M/β-CD; Jsc was obtained as a function of light intensity.

Section 3. Thin film characterization

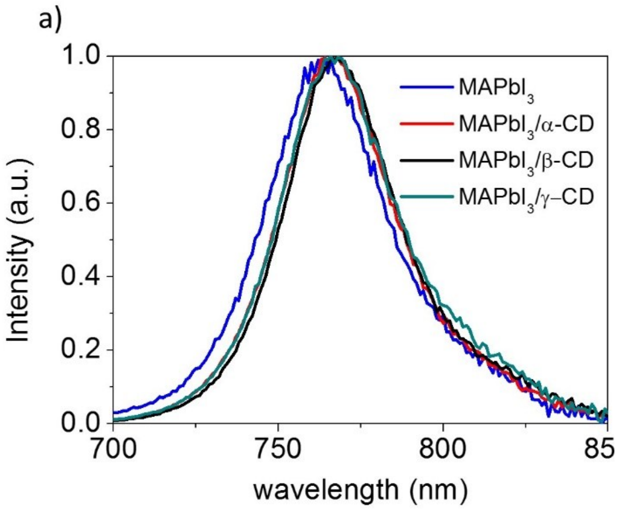

Figure S8. Steady-state photoluminescence (PL) of $MAPbI_3$ 1M and $MAPbI_3$ 1M/CDs films.

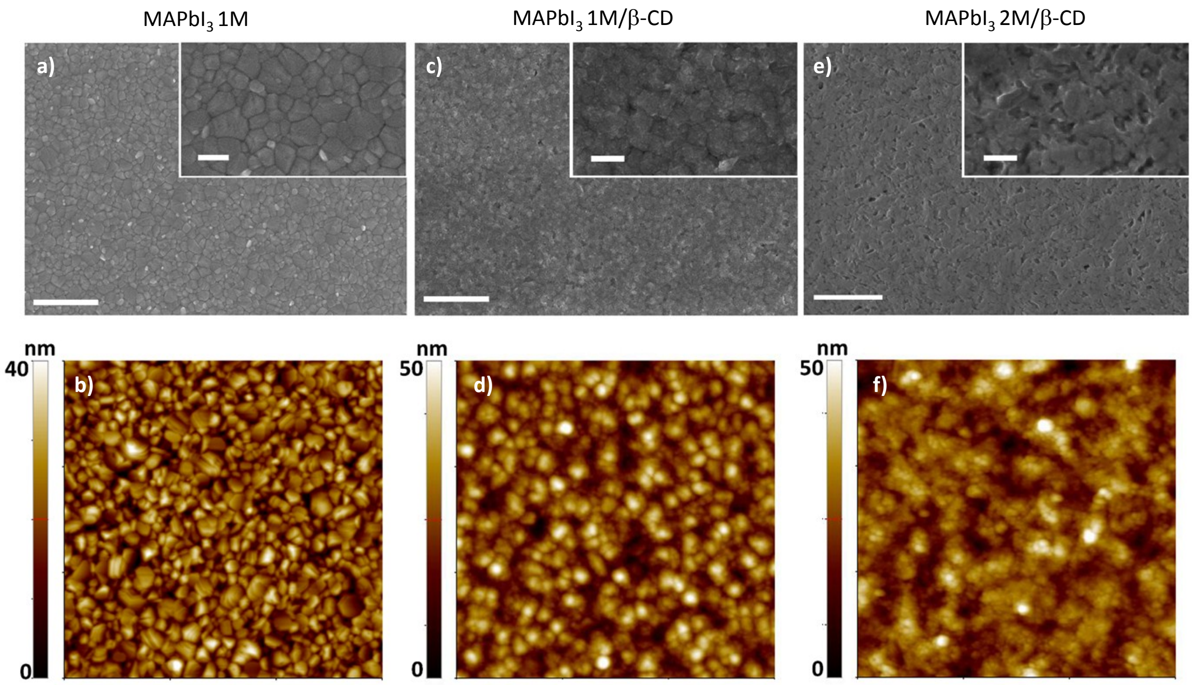

Figure S9. Scanning Electron Microscopy (SEM) images of a) $MAPbI_3$ 1M, c) $MAPbI_3$ 1M/β-CD, e) $MAPbI_3$ 2M/βCD films; Atomic Force Microscopy (AFM) images of b) $MAPbI_3$ 1M, d) $MAPbI_3$ 1M/β-CD, f) $MAPbI_3$ 2M/β-CD films; SEM image shows more compact and merged grains for β–CD/perovskite material, slightly bigger in size withstanding a film roughness below 10 nm (AFM). Variations in morphology could be attributed to the MAI disaggregation by β-CDs (observed with NMR) or to the improved solubilization of PbI_2 (Figure 1d) that has a two-fold effect: enhancing the propensity of PbI_2 to interact with MAI and reducing the size of PbI_2 aggregates in solution. The perovskite self-assembly on films is guided by PbI_2 aggregates that act as nucleation seeds,[13,14] then evolving in a very different morphology when β-CD is added. AFM are (3μmx3μm) and SEM scale bar are 1 μm and 200 nm for the zoom Rq=6.6nm ($MAPbI_3$); Rq=9.6nm ($MAPbI_3$ 1M/β-CD); Rq=8.5 ($MAPbI_3$ 2M/βCD).

Section 4. Advanced structural characterization

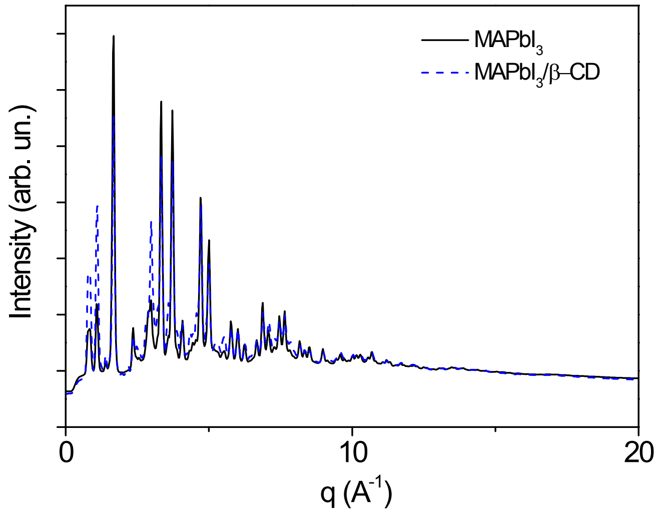

Figure S10. Synchrotron XRD patterns from $MAPbI_3$ 1M and $MAPbI_3$ 2M/β-CD powders (XPD).

Table S7. Results from the X-ray synchrotron characterization. Characteristic feature of the $MAPbI_3$ structural models and relative amount of the PbI_2-DMSO-MAI intermediate crystal phase.

	**$MAPbI_3$ crystal phase in the space group I 4/m c m**	
**Samples**	**Torsion angle I-Pb-Pb-I along c (°)**	**DMSO/PbI_2 MAI fraction (%)**
Initial model	20.1	--
$MAPbI_3$	14.8	37
$MAPbI_3$/ β-CD	16.7	14

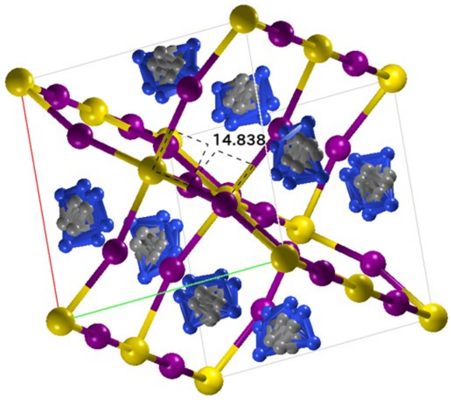

Figure S11. Derived 3D structure of $MAPbI_3$ perovskite (no-CD sample), highlighting the torsion angle that is significantly changing in the sample containing β-CD.

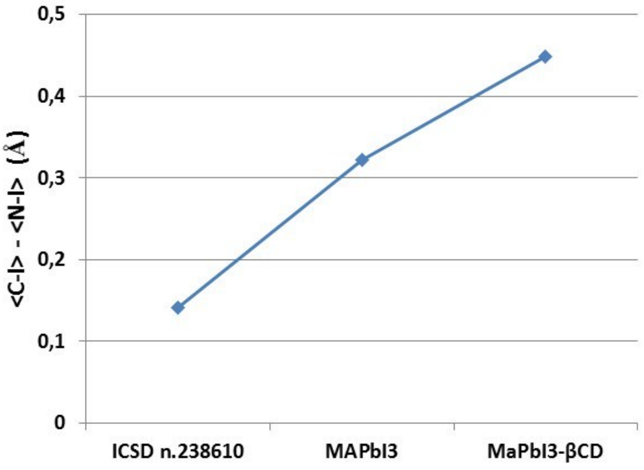

Figure S12. Difference between average C-I and N-I distances, calculated by considering the four first neighboring I^- anions, and one of the possible C-N equivalent configurations shown in Fig. 6 (main text). Structural parameters are calculated from the tetragonal $MAPbI_3$ structure (ICSD database n.238610), and the models obtained by the PDF refinement of the $MAPbI_3$ and the $MAPbI_3$-CD samples.

References

1 M. M. Lee, J. Teuscher, T. Miyasaka, T. N. Murakami, H. J. Snaith, *Science*, 2012, **338**, 643-647.

2 A. P. Hammersley, S. O. Svensson, M. Hanfland, A. N. Fitch, D. Hausermann, *High Pressure Research*, 1996, **14**, 235-248.

3 R. Caliandro, D. B. Belviso, *Journal of Applied Crystallography*, 2014, **47**, 1087-1096.

4 M. Morháč, V. Matoušek, *Applied Spectroscopy*, 2008, **62**, 91-106.

5 P. Juhas, T. Davis, C. L. Farrow, S. J. L. Billinge, *Journal of Applied Crystallography*, 2013, **46**, 560-566.

6 C. L. Farrow, P. Juhas, J. W. Liu, D. Bryndin, E. S. Bozin, J. Bloch, T. Proffen, S. J. Billinge, *Journal of Physics: Condensed Matter*, 2007, **19**, 335219.

7 C. C. Stoumpos, C. D. Malliakas, M. G. Kanatzidis, *Inorganic Chemistry*, 2013, **52**, 9019-9038.

8 S. Ito, S. Tanaka, H. Nishino, *The Journal of Physical Chemistry Letters,* 2015, **6**, 881-886.

9 Y. Yamada, T. Yamada, L. Q. Phuong, N. Maruyama, H. Nishimura, A. Wakamiya, Y. Murata, Y. Kanemitsu, *Journal of the American Chemical Society,* 2015*,* **137**, 10456-10459.

10 M. C. Burla, R. Caliandro, M. Camalli, B. Carrozzini, G. L. Cascarano, C. Giacovazzo, M. Mallamo, G. Mazzone, R. Polidori, *Journal of Applied Crystallography*, 2012, **45**, 357-361.

11 A. Giuri, S. Masi, S. Colella, A. Kovtun, S. Dell'Elce, E. Treossi, A. Liscio, C. Esposito-Corcione, A. Rizzo, A. Listorti, *Advanced Functional Materials,* 2016, **26**, 6985-6994.

12 N. J. Jeon, J. H. Noh, Y. C. Kim, W. S. Yang, S. Ryu, S. Il. Seok, *Nature Materials,* 2014*,* **13**, 897-903.

13 R. Mastria, S. Colella, A. Cualtieri, A. Listorti, G. Gigli, A. Rizzo, *Nanoscale*, 2017, **9**, 3889-3897.

14 K. Yan, M. Long, T. Zhang, Z. Wei, H. Chen, S. Yang, J. Xu, *Journal of the American Chemical Society*, 2015, **137**, 4460-4468.
